# Mammalian RNA switches: Molecular rheostats in gene regulation, disease, and medicine

**DOI:** 10.1016/j.csbj.2019.10.001

**Published:** 2019-10-24

**Authors:** Kadiam C. Venkata Subbaiah, Omar Hedaya, Jiangbin Wu, Feng Jiang, Peng Yao

**Affiliations:** a Aab Cardiovascular Research Institute, Department of Medicine, University of Rochester School of Medicine & Dentistry, Rochester, NY 14586, United States; b Department of Biochemistry & Biophysics, University of Rochester School of Medicine & Dentistry, Rochester, NY 14586, United States; c The Center for RNA Biology, University of Rochester School of Medicine & Dentistry, Rochester, NY 14586, United States; d The Center for Biomedical Informatics, University of Rochester School of Medicine & Dentistry, Rochester, NY 14586, United States

**Keywords:** Disease, Gut microbiota, Medicine, Metabolite, MicroRNA, Mitochondria, Protein-directed RNA switch, Riboswitch, RNA binding protein, Translational control

## Abstract

Alteration of RNA structure by environmental signals is a fundamental mechanism of gene regulation. For example, the riboswitch is a noncoding RNA regulatory element that binds a small molecule and causes a structural change in the RNA, thereby regulating transcription, splicing, or translation of an mRNA. The role of riboswitches in metabolite sensing and gene regulation in bacteria and other lower species was reported almost two decades ago, but riboswitches have not yet been discovered in mammals. An analog of the riboswitch, the protein-directed RNA switch (PDRS), has been identified as an important regulatory mechanism of gene expression in mammalian cells. RNA-binding proteins and microRNAs are two major executors of PDRS via their interaction with target transcripts in mammals. These protein-RNA interactions influence cellular functions by integrating environmental signals and intracellular pathways from disparate stimuli to modulate stability or translation of specific mRNAs. The discovery of a riboswitch in eukaryotes that is composed of a single class of thiamine pyrophosphate (TPP) suggests that additional ligand-sensing RNAs may be present to control eukaryotic or mammalian gene expression. In this review, we focus on protein-directed RNA switch mechanisms in mammals. We offer perspectives on the potential discovery of mammalian protein-directed and compound-dependent RNA switches that are related to human disease and medicine.

## Graphical abstract

**Figure:**
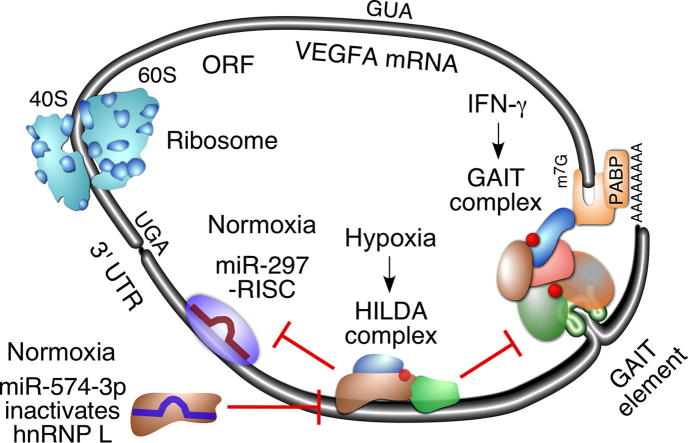

## 1 Overall introduction: The riboswitch and mammalian protein-directed RNA switch

Ribonucleic acid (RNA) plays a versatile role in translating genetic codon information from DNA genomes into functional proteins for living organisms. The alteration of RNA structure by environmental signals via the action of riboswitches is recognized as an essential mechanism that regulates gene expression in prokaryotes and eukaryotes [1], [2], [3]. The riboswitch is an RNA regulatory element of a corresponding mRNA. It binds to a small molecule and causes a structural change in the RNA, independent of protein binding, thereby regulating mRNA transcription, splicing, or translation [1]. In general, a riboswitch contains two parts, namely, an RNA “aptamer”, and an “expression platform”. The RNA “aptamer” is a structural element generally situated in the 5'UTR of the regulated mRNA, and is a biosensor for direct binding of the ligand. Upon ligand binding, this RNA aptamer induces a conformational change immediately downstream, in a region of the RNA called the “expression platform”. This alteration inhibits gene transcription or initiation of the translation of transcripts that encode proteins (synthase or transporter), which regulate the concentration of the ligand in the cell [2], [4], [5].

In prokaryotes, about 40 families of riboswitches have been identified that respond to diverse effectors, including coenzymes, nucleotide derivatives, amino acids, and signaling molecules [6]. There are also classes of temperature-, metal-, pH-, and ion-sensing regulatory RNAs [7], [8], [9], [10]. Ligand-sensing RNAs employ diverse mechanisms to interact with the transcriptional or translational machinery to control gene expression [11]. For riboswitches that function at the transcriptional level, effector–RNA binding stabilizes intrinsic terminator or anti-terminator hairpins to inhibit or activate transcription [11]. In prokaryotes, translation initiation requires the small ribosomal subunit to recognize ribosome-binding sites. Riboswitches that work at the translational level function by masking or exposing ribosome-binding sites to repress or activate translation [11].

To date, only one type of riboswitch, thiamine pyrophosphate (TPP), has been reported for eukaryotes, in fungi [12] and plants [13], [14]. In fungi, this riboswitch resides within an intron and modulates alternative splicing, while in plants it is located in the 3′-untranslated region (3′UTR) and regulates alternative polyadenylation and mRNA stability. Hence the current dogma is that riboswitches exist only in bacteria, fungi, and plants [15]. One likely reason is that because bacterial genomes are highly compact, their DNA contains genetic information that is capable of directing the self-regulation of genes, allowing for efficient adaptation to changes in environmental metabolites in order to maintain species survival. An important, long-unanswered question in the field of RNA biology is whether riboswitches exist in mammals [11], [16].

More than a decade has elapsed since the TPP riboswitch sequences were first identified in eukaryotes [17]. The discovery of a single class of riboswitch in eukaryotes suggests that additional ligand-sensing RNAs may be present to control eukaryotic gene expression. Structural changes in noncoding RNAs in vertebrates have been reported in the cationic amino acid transporter-1 (Cat-1) internal ribosome entry site (IRES) in response to amino acid starvation [18]. Changes have also been found in the Apaf1 IRES, which is remodeled for ribosome entry [19]. In addition, an RNA thermosensor has been discovered during heat shock response in mammalian cells [20]. However, in none of these responses has the presence of metabolite-sensing riboswitches been established.

In recent years, compound protein-directed RNA switches (PDRSs) have been recognized as emerging translational regulators of gene expression in complex eukaryotic organisms [21], [22]; these serve as riboswitch analogs and add another layer of complexity to the network of gene regulation. The PDRS is driven by mutually exclusive interactions of two (or more) sets of RNA-binding proteins (RBPs) or their complexes, with the same or adjacent *cis*-acting RNA elements. The interactions involve either structural remodeling [23] or physical competition [24]. The PDRSs are either structural or linear, each mediated by their specific class of *trans*-acting regulators. Structural PDRSs involve secondary structure-recognizing RBPs, such as double-stranded RNA-binding proteins (DRBP76, RNA helicases) and the Gamma-interferon Activated Inhibitor of Translation (GAIT) complex [21], [25], [26], whereas linear PDRSs are directed by sequence-specific factors, such as heterogeneous nuclear ribonucleoproteins (hnRNPs) and miRNAs [22], [23].

In one example, PTBP/hnRNP I binds to CU-rich sequence and disrupts the local secondary structure, such as the stem-loop, thereby exposing the RNA to targeting by the miRNA-induced silencing complex [22]. In another example, posttranscriptional modification of N(6)-methyladenosine in mRNAs disrupts the Watson-Crick base pair of A-U and increases the binding affinity of hnRNP C to its target mRNAs [27]. These RNA-protein interactions integrate environmental or intracellular signals from disparate stimulatory conditions to regulate gene expression at the post-transcriptional level and alter cellular function. PDRSs can either inhibit or reversibly activate mRNA translation, depending on the state of environmental stress.

There are well characterized PDRSs in bacteria that have been described in previous reviews [28], [29]. Here, we focus on mammalian protein-directed RNA switch mechanisms and provide perspectives on potential PDRSs and riboswitch-like RNA switches in mammals. We speculate that metabolic enzymes, ribosomes, and pathogenic and pharmacological small molecules drive these hypothetical mammalian RNA switches, which may modulate metabolic homeostasis, disease progression, and beneficial or side effects of drugs.

## 2 Protein-directed RNA switches in mammals

### 2.1 hnRNP L-directed RNA switches activate mRNA translation

Transcript-selective translational control is generally mediated by interaction of RNA-binding proteins or complexes and sequence/structural elements in 5′ or 3′UTR of target transcripts [30], [31], [32]. This control mechanism facilitates co-regulation of functionally-related transcripts bearing common elements in a post-transcriptional regulon [33], [34]. Recently, an additional layer of complexity has been recognized in which pairs of elements can act as condition-dependent RNA switches.

**Fig. 1:**
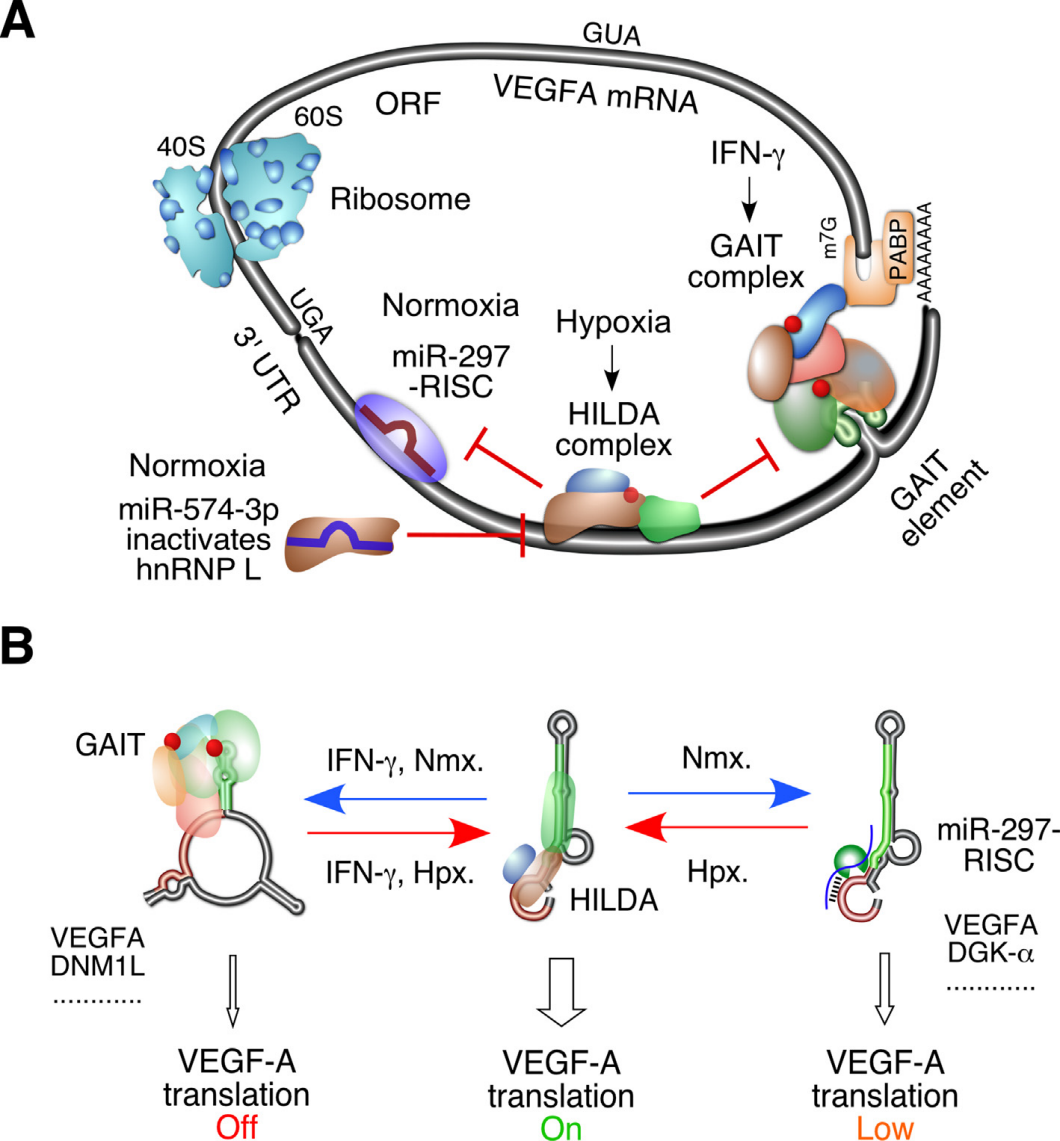
Protein-directed RNA switch in the 3′UTR of VEGFA and other transcripts. (A) GAIT- and miR-297-RISC-dependent, hnRNP L-containing HILDA complex-directed VEGFA RNA switch. miR-574 acts as an RNA decoy to negatively regulate the VEGFA RNA switch. (B) The secondary structure of the 125-nt VEGFA hypoxia stability region (HSR) in the translation-permissive conformation (middle). The GAIT element (green) and hnRNP L binding CA-rich element (CARE, red) are highlighted. The GAIT complex binds to the GAIT element and represses *VEGFA* mRNA translation under IFN-γ stimulus; while the hnRNP L-bearing HILDA complex binds to CARE and prevent GAIT complex binding and restores *VEGFA* mRNA translation under hypoxic condition (Left). miR-297-RISC binds to CARE of *VEGFA* mRNA and inhibits its translation in normoxia; while the HILDA complex binds to CARE and blocks the interaction of miR-297-RISC and activates *VEGFA* mRNA translation in hypoxia (Right). (For interpretation of the references to colour in this figure legend, the reader is referred to the web version of this article.)

Our previous studies have shown that a vascular endothelial growth factor-A (VEGFA) PDRS controls translation of *VEGFA* mRNA in myeloid cells that respond to hypoxic and inflammatory signals (Fig. 1A). IFN-γ-activated GAIT complex mediated silencing of VEGFA translation [35]. The GAIT complex is assembled by the central RNA-binding protein glutamyl-prolyl-tRNA synthetase (EPRS) [36], [37], ribosome protein L13a (RPL13a), and two regulatory proteins synaptotagmin-binding cytoplasmic RNA interacting protein (NSAP1) and GAPDH [38]. The GAIT-mediated translational repression of VEGFA is overcome during hypoxia [23]. The translation state is regulated by a binary, mutually exclusive conformational switch in the *VEGFA* mRNA 3′UTR, directed by IFN-γ and hypoxia-dependent binding of the GAIT and hnRNP L (heterogeneous nuclear ribonucleoprotein L)-bearing complexes, respectively [23]. hnRNP L is a CA-rich element (CARE)-binding protein that binds to RNA regions containing nearby CACA, CAUA, or ACAC motifs via RNA recognition motif domains [39]. hnRNP L plays a critical role in nuclear pre-mRNA splicing and cytoplasmic mRNA switching in different cellular compartments [21], [40], [41]. In normoxia, hnRNP L is primarily localized in the nucleus, with a small fraction in the cytoplasm [24]. During hypoxic stress, a substantial amount of hnRNP L is translocated to the cytoplasm upon phosphorylation by the Src kinase and associates with two other RBPs to form the trimeric Hypoxia-Induced hnRNP L-DRBP76-hnRNP A2/B1 (HILDA) complex [21], [42]. The HILDA complex binds to a CARE in the *VEGFA* mRNA and alters the RNA secondary structure to abolish binding of the GAIT complex to the hypoxia stability region (HSR), thereby activating *VEGFA* mRNA translation [21], [24] (Fig. 1B). The double-stranded RNA binding protein DRBP76 plays a critical role in disrupting the stem-loop-containing GAIT RNA element and promoting the formation of a long AU-rich double-stranded RNA conformation [21]. Our global bioinformatic analyses have revealed several human transcripts bearing both GAIT- and hnRNP L-binding elements in their 3′UTRs. These, together with the elements in the *VEGFA* 3′UTR, modulate the translation of a family of mRNAs via PDRS, including VEGFA, DNM1L, and PHF21A among others (Fig. 1B) [42].

Another examplar RNA switch is mediated by hnRNP L and miRNA-induced silencing complex (miRISC) [24]. miRISC generally contains a central RNA binding protein, Argonaute (e.g., Ago2), that binds to a single stranded miRNA. The miRNA acts as a guide RNA for the RISC to recognize and bind complementary mRNA transcript. Once bound, the Argonaute protein catalyzes the cleavage or represses the translation of the target mRNA [43]. In the absence of IFN-γ stimulus in myeloid cells, miR-297-RISC binds to CARE in the HSR of *VEGFA* mRNA 3′UTR and inhibits translation. Under hypoxic stress, hypoxia-induced cytoplasmic hnRNP L can block miR-297-RISC binding to the CARE in the HSR (Fig. 1) [24].

In addition, we have recently reported several other mRNA transcripts that contain miR-297-dependent, hnRNP L-directed RNA switch elements, including *MKLN1*, *EIF5*, and *CDK6* [42]. Similarly, miR-297 and hypoxia-activated hnRNP L were shown to regulate translation of diacylglycerol kinase-alpha (*DGK-α*) mRNA in human glioma cells via the same mechanism reported for the *VEGFA* mRNA switch, thereby promoting glioblastoma progression [44]. Moreover, translational regulation by hnRNP L via potential competition between hnRNP L and miRNA binding was observed for a cohort of target mRNA 3′UTRs in Hela cells [40]. These findings suggest that hnRNP L-directed human RNA switches are broadly present across multiple cell types.

The VEGFA PDRS represents a founding member of riboswitch-like RNA switches in mammals that respond to physiological or pathological stimuli to control gene expression. Three unique features distinguish it from riboswitches in bacteria: (a) Bacterial riboswitches sense physiological signals by direct binding of the effector molecule, without requiring signal “interpretation” by a regulatory protein. For the human VEGFA PDRS, the sensor and switch mechanisms are protein-mediated [21], [23]. (b) While bacterial riboswitches are located in the 5' leader [1], the *VEGFA* PDRS is located in the mRNA 3'UTR [21], [23]. (c) The *VEGFA* PDRS uses a novel switching mechanism to integrate the response to two different physiological stimuli. The *VEGFA* PDRS is a single RNA with interdependent binding sites in two distinct, ligand-responsive conformational states (Fig. 1B). This multi-input integration of signals is advantageous when an organism is regulating gene expression and maintaining cellular homeostasis in response to combinations of stress signals.

In summary, we have described a multi-protein, multi-element, condition-dependent, conformational switch in the 3′UTR of human *VEGFA* mRNA among other transcripts – the first vertebrate RNA switch that controls translation. These novel RNA elements will help us understand how pairs (or larger clusters) of RNA elements in noncoding regions of mRNAs, and their cognate interacting proteins, integrate physiological and pathological stimuli to regulate translation in mammalian cells.

### 2.2 miRNA functions as an RNA decoy to regulate the VEGFA RNA switch

Now that the hnRNP L-dependent PDRSs have been identified, next big question is how are these RNA switches regulated? Based on the molecular mechanisms of the VEGFA RNA switch described above, we believe that regulation of target mRNA translation by hnRNP L-directed PDRS is controlled by five parameters: (a) cell-type- or tissue-specific expression of a spectrum of target mRNAs; (b) cytosolic concentration of a target mRNA under normoxic or hypoxic conditions; (c) cytosolic concentration of hnRNP L, determined by hypoxia-induced cytoplasmic translocation; (d) hnRNP L binding affinity for a target mRNA CARE; and (e) availability of hnRNP L to interact with target mRNAs that could be regulated by “sponging” factors such as miRNAs. Recent findings from multiple laboratories indicate that miRNAs can function as sequence-specific RNA decoys and modulate RBP function via seed sequence-independent interaction with RBPs [45], [46], [47], [48]. Similar RBP-miRNA interactions could conceivably work in the opposite direction, through RBP-mediated inhibition of target miRNA function [47], [49].

Recently, we have elucidated the regulation of the *VEGFA* mRNA switch by miR-574-3p via interaction with hnRNP L under pathophysiological conditions [47]. We have shown that in normoxia, moderately expressed CA-rich miR-574-3p binds a small amount of cytoplasmic hnRNP L and inhibits the *VEGFA* mRNA switch (Fig. 1A). We have also demonstrated that hypoxia-induced cytoplasmic hnRNP L binds miR-574-3p and inactivates its tumor-suppressive function by preventing its loading onto the miRISC. The mutual regulation of the activities between miR-574-3p and hnRNP L does not depend on the 5′ terminal seed sequence, but the CA-rich sequence of “CACACACCCACA” in the 3′ end of the miRNA. Intriguingly, miR-574-3p is stable when bound by hnRNP L and unassociated with Ago2, suggesting that hnRNP L may protect miR-574-3p from miRNA decay pathways such as Tudor-SN-mediated endonucleolytic cleavage at CA and UA dinucleotides in miRNAs [50]. Finally, as a potential therapeutic approach, we have shown that in hypoxia, overexpressed miR-574-3p acts as a decoy for hnRNP L, reverses the *VEGFA* mRNA switch, and inhibits cancer cell growth in a xenograft mouse tumor model. Together, these findings establish for the first time a condition-dependent two-way regulation based on miRNA-RBP interaction and provide a proof-of-principle rationale for developing miRNA-based, hnRNP L-targeted therapeutic strategies against cancer [51], [52].

### 2.3 hnRNP E2-mediated CEBPA RNA switch silences mRNA translation

**Fig. 2:**
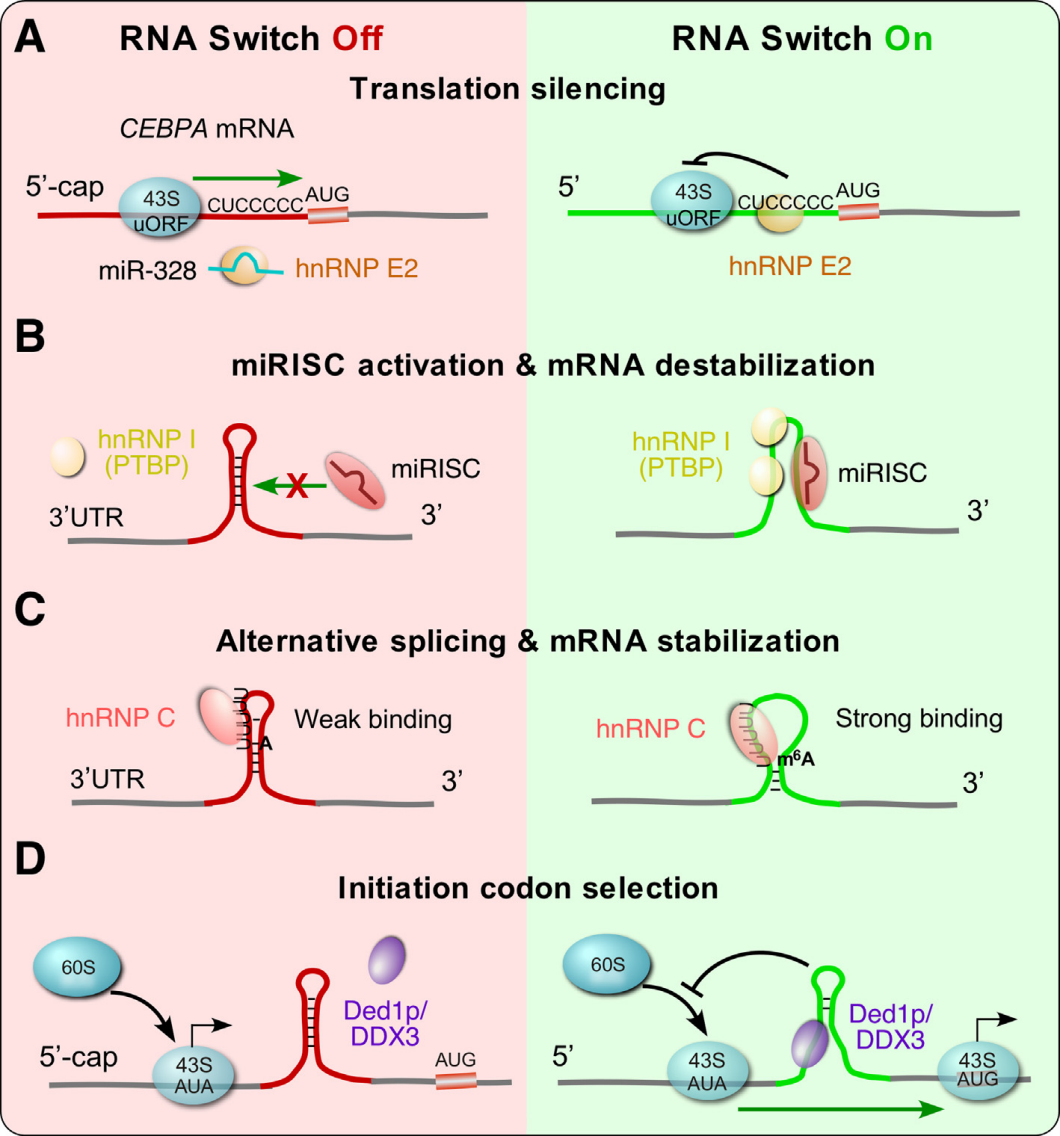
Schematic model of mammalian protein-directed RNA switches. Four types of human PDRSs are shown. (A) 5′UTR-dependent, hnRNP E2-mediated blockage of 43S pre-initiation complex scanning and miR-328-driven inactivation of hnRNP E2 and translational activation of *CEBPA* mRNA during myeloid cell differentiation. (B) miRNA-dependent, PTBP-directed RNA switches during fibroblast-to-neuron *trans*-differentiation. (C) m^6^A-dependent, hnRNP C-directed RNA switches in HEK293T cells. (D) Ded1p/DDX3-mediated structural switch of 5′UTR regulates translation initiation codon selection in yeast.

In addition to hnRNP L-directed RNA switches that activate mRNA translation, another hnRNP-mediated RNA switch mechanism has been reported to silence mRNA translation during the differentiation process of myeloid progenitor cells (Fig. 2A) [45]. A poly(rC)-binding protein, hnRNP E2, typically binds to a cytosine (C)-rich *cis*-acting RNA element (CUCCCCC) located between an upstream open reading frame (uORF) and the main ORF in the 5′UTR of the target mRNA, *CEBPA* (CCAAT enhancer binding protein alpha). This interaction may block scanning of the 43S pre-initiation complex through the 5′UTR, prevent the formation of a mature ribosome, and repress mRNA translation. An abundantly expressed, C-rich miR-328 acts as an RNA decoy to bind and capture hnRNP E2. The formation of miR-328-hnRNP E2 complex releases hnRNP E2 from the 5′UTR C-rich RNA element in *CEBPA* mRNA, thereby restoring the translation of CEBPA and driving myeloid cell differentiation. In contrast, loss or reduction of miR-328 expression in blast crisis chronic myelogenous leukemia permits hnRNP E2-mediated translational silencing of *CEBPA* mRNA, blocks myeloid cell differentiation, and leads to oncogenic growth of blood cells.

Putting these findings together, we think that miRNAs not only act as a ligand to directly participate in the PDRS (e.g., miR-297), but also can bind and inactivate RBPs to regulate PDRS (e.g., miR-574-3p and miR-328). In the future, it will be interesting to test whether two or more miRNAs could synergistically conduct or regulate PDRS, and whether manipulating concentrations of those miRNAs could modulate RNA switches and reverse pathogenic phenotypes *in vivo*.

### 2.4 PTBP-directed RNA switches regulate miRISC activation and mRNA destabilization

In contrast with the role of miRNAs in sponging RBPs and regulating RNA switches, RBPs have been shown to inhibit or promote the activity of miRNAs and the stability of their target mRNAs. Polypyrimidine tract binding protein PTBP (also known as PTB or hnRNP I) is well-characterized as a splicing repressor through binding to CU-rich elements in mRNA precursors [53], [54]. In addition to regulating splicing, PTBP protein has also been reported to regulate mRNA stability under different physiological conditions [55], [56], [57]. One possible molecular mechanism is the PTBP-mediated RNA structural change that increases the accessibility of other RNA binding factors such as miRNAs (Fig. 2B). For instance, PTBP cooperates with miR-221 to regulate the translation of *AdipoR1* (Adiponectin receptor protein 1) mRNA during muscle differentiation and in obesity [58]. PTBP and miR-221 bind to two adjacent regions in *AdipoR1* mRNA 3′UTR. These two binding sites form a double-stranded stem-loop structure in the absence of PTBP and miR-221. PTBP binding destroys the stem-loop structure and facilitates a linear RNA conformation, thus allowing miR-221-RISC to bind to its target site and inhibit the translation of *AdipoR1* mRNA [58]. We term this RNA structural change as a miRNA-dependent, PTBP-directed RNA switch.

Another example of this type of RNA switch is driven by PTBP for maintenance of specific cell lineages [22]. PTBP competes directly with miR-124 to bind the 3′UTR of its target mRNAs synaptonemal complex protein 1 (*SCP1*) and REST corepressor (*CoREST*), whose encoded proteins play essential roles in preventing fibroblast-to-neuron transition [59], [60]. These two proteins are components of the REST (RE1-silencing transcription factor) complex and repress the expression of a large number of neuronal genes. In addition, PTBP promotes the activities of some miRNAs on target mRNAs through binding to the adjacent sequence of miRNA targeting sites and changing the local secondary structure to facilitate miRNA binding, e.g., let-7b and miR-181b binding to their common target mRNA glucosamine-6-phosphate deaminase 1 (*GNPDA1*) [22]. Intriguingly, knockdown of a single RBP, PTBP, or in combination with a second RBP, nPTB (neural PTB), drives *trans*-differentiation of fibroblasts to functional neurons *in vitro* [22], [61].

Taken together, these two studies suggest that PTBP exerts its non-splicing regulatory function to regulate miRISC activity in cytoplasm under different pathophysiological conditions. Furthermore, PTBP-dependent PDRS provides a molecular model for understanding co-regulation between different RNA-binding proteins and miRNAs. It has been observed that a large number of miRNAs are localized in the nucleus [62]. Thus, it remains an open question whether PTBP drives an RNA switch in the nucleus and regulates alternative splicing by influencing potential miRNA-dependent binding of precursor mRNAs.

### 2.5 Nucleic acid modification promotes RNA switch to modulate alternative splicing and mRNA stabilization

An emerging nucleic acid modification that activates the RNA switch is N(6)*-*methyladenosine (m^6^A) RNA methylation (Fig. 2C), which is one of the universally prevalent RNA modifications present in eukaryotic mRNA, tRNA, rRNA, and snRNA, as well as some long, non-coding RNAs (lncRNA) [63], [64], [65]. The m^6^A modification is directed at RRACH (R = G/A, H = U/A/C) motifs by a methyltransferase complex bearing METTL3 and METTL14 (m^6^A writers) [66]. As a dynamic and reversible modification, m^6^A methylation can be removed by two major demethylases: fat mass and obesity-associated protein (FTO) and alkB homolog 5 (ALKBH5) [67], [68]. This m^6^A modification regulates RNA stability, splicing, export, and translation efficiency [69], [70]. m^6^A is commonly believed to function by interacting with YTH-domain-containing proteins (m^6^A readers) [69].

m^6^A modification, in addition to being recognized directly by m^6^A reader proteins, has also been shown to mediate RNA-protein interactions. It increases the accessibility of its surrounding RNA sequences through altering local RNA secondary structures, a process termed m^6^A-directed RNA switch or m^6^A switch [27]. m^6^A modification was found to mediate interactions between heterogeneous nuclear ribonucleoprotein C (hnRNP C), an RNA binding protein that facilitates mRNA processing, and a U_5_-containing hairpin present in a lncRNA, namely, metastasis-associated lung adenocarcinoma transcript 1 (MALAT1) [27], [71]. In this case, m^6^A modification at position A2577 inside an “AGG” sequence weakens its base pairing with the third U in the opposite U_5_-tract inside the stem-loop hairpin. Thus, the first three Us in the U_5_-tract become more accessible to hnRNP C, which leads to an eightfold increase in the hnRNP C binding affinity [27]. By using a combination of anti-m^6^A immunoprecipitation (MeRIP)-Seq and photoactivatable-ribonucleoside-enhanced crosslinking and immunoprecipitation (PAR-CLIP) in normal and global m^6^A reduction conditions, the authors identified around 2800 high-confidence m^6^A-dependent RNA switches in hnRNP C-binding RNAs [27]. Further biochemical and biophysical analyses using Förster resonance energy transfer and nuclear magnetic resonance demonstrate that m^6^A destabilizes the U_5_-tract of the stem-loop and converts the structure of an m^6^A-containing hairpin towards the induced conformation in the hnRNP C–RNA complex [71]. Gene expression analyses suggest that the m^6^A RNA switch and hnRNP C binding activity regulate mRNA splicing, maturation, and stability. This mechanism was uncovered in a model cell system HEK293T human embryonic kidney cell line, but may be generalizable to other cell types. Besides hnRNP C, another m^6^A-directed RNA switch has also been found to mediate interactions between a purine-rich secondary structure in MALAT1 and low-complexity Arg-Gly-Gly repeats of hnRNP G [72]. The consensus motif for hnRNP G-binding target RNAs, AGRAC (R = A/G), partially overlaps with the classical m^6^A motif.

Collectively, these studies suggest that the m^6^A modification-dependent RNA conformational switch may be a universal molecular mechanism to modulate the accessibility of RBPs to specific RNA and influence their function. Further investigations should explore whether other RBP-mediated RNA switches are regulated by m^6^A or other modified nucleic acids (e.g., m^5^C) [73].

### 2.6 Ded1p/DDX3 mediates RNA switches for initiation codon selection

An 5′ upstream ORF (5′ uORF) is defined as an open reading frame located in 5′UTR of mRNAs and out-of-frame with the main coding sequence. In general, translation of 5′ uORF reduces the protein expression of the downstream main ORF (mORF) [74]. To date, the *trans*-acting factors have not been well characterized during the regulation of the translation of uORF and mORF.

Previous studies have shown that the presence of a secondary structure downstream of an alternative AUG or a near-cognate initiation codon facilitates alternative translation initiation at uORF [75]. More recent experiments confirmed this observation *in vivo* in yeast [76]. The DEAD-box RNA helicase Ded1p, the yeast homolog of mammalian DDX3, is known to participate in RNA processing and translation regulation. Dr. Jankowsky’s laboratory has shown that introduction of a T408I mutation in the yeast Ded1p protein abolishes its unwinding activity. Then by using ribosome profiling together with dimethyl sulfate mutational profiling with sequencing (DMS-MaPseq), they were able to show more ribosomal stalling upstream of RNA secondary structures in the 5′UTR of mRNAs in the presence of the T408I mutant helicase, compared to the wild-type protein. Subsequently, translation initiation occurred at alternative upstream translation initiation sites [76] (Fig. 2D). They also highlight that during meiosis, Ded1p expression decreases during anaphase II relative to the vegetative state, modulating translation initiation and indicating a potential role for this mechanism *in vivo*. They raise an intriguing question of whether this mechanism could be used by the eukaryotic cell as a riboswitch-like mechanism. This question was motivated by the findings that Ded1p/DDX3 switches between repression and initiation of translation, by either the formation of mRNP granules or their resolution, followed by translational activation upon Ded1p/DDX3-mediated ATP hydrolysis [77]. In addition, Ded1p/DDX3 and other RNA helicases could sense ATP depletion through the elevated levels of AMP, which abrogates their RNA binding and unwinding ability [78].

In summary, Ded1p/DDX3 unwinds RNA secondary structures in 5′UTR of mRNAs and controls translation initiation codon selection, thereby extending the N-terminal sequence of a large number of proteins. In the future, the function and mechanism of action of DDX3 protein in remodeling 5′UTR local secondary structure and regulating translation initiation codon selection need to be studied in mammals, compared to yeast.

## 3 Perspective on the potential discovery of human protein- and chemical compound-dependent RNA switches

The current strategy to discover bacterial riboswitches has relied on bioinformatic predictions based on sequence and structure conservation. Many laboratories have searched computationally for bacterial riboswitch sequences in the human genome without a productive outcome, possibly because the riboswitch function in bacteria has no corollary in humans. If there are human riboswitch-like RNA switches, such as an ATP sensor, they are likely to be different in sequence and structure from switches in lower order species. The challenge is to shift this paradigm by establishing proof that riboswitch-like RNA aptamers are present in mammals, including humans, where they function in both healthy and disease states.

Dr. Luptak’s laboratory used bioinformatic predictions and *in vitro* systematic evolution of ligands by exponential enrichment (SELEX) to identify human ATP-dependent RNA aptamers as components of putative riboswitches [79], [80]. However, these have not been studied in cells, and they are buried deep inside intronic regions of several pre-mRNAs whose mRNAs and encoded proteins have no apparent functional relationship with ATP. These factors confound the significance of Luptak’s finding and its generalizability to mammals. Besides, a mammalian RNA thermosensor has been reported from Dr. Nudler’s laboratory [20]. Shamovsky et al. discovered that heat-shock transcription factor 1 (HSF1) activation during heat shock stress was mediated by a unique ribonucleoprotein complex containing a non-coding RNA heat shock RNA-1 (HSR1) and an eukaryotic translation elongation factor eEF1A1. A conformational switch occurs in HSR1 in response to heat shock (43 °C) and is required for enhanced interaction with eEF1A1 and HSF1 activation. However, the molecular mechanism of the HSR1 RNA structural change has not been elaborated [81], including the role of eEF1A1 binding or temperature change in the HSR1 conformational switch, and information about the structural switch in the two identified 5′ terminal domains of HSR1 [20].

**Fig. 3:**
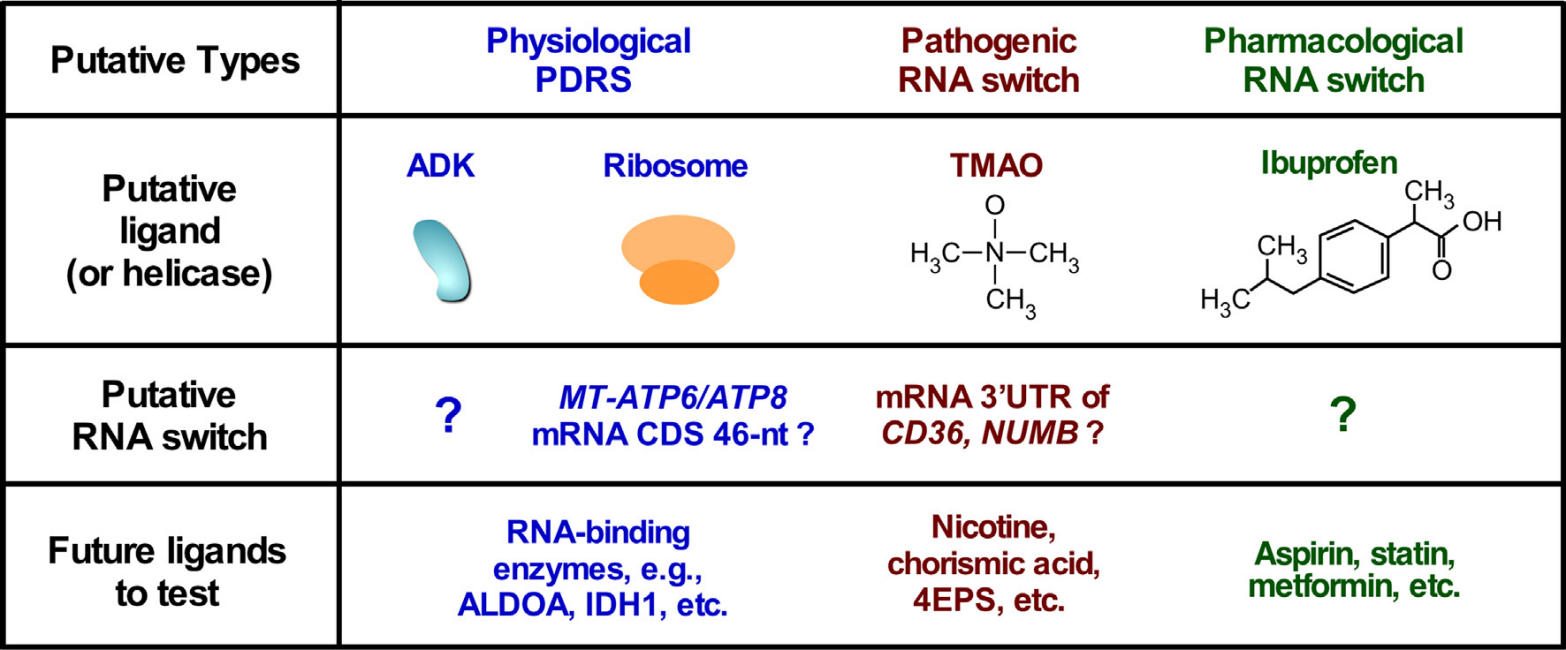
Four types of potential mammalian RNA switches. Putative physiological (two types), pathogenic, and pharmacological human RNA switches and their putative ligands are proposed. See text for details. A ribosome is an RNA helicase that unwinds the putative 46-nt stem-loop located in ATP8/ATP6 mRNA coding sequence (CDS). Nicotine: an alkaloid produced in tobacco and an environmental compound from smoking. Chorismic acid: an intermediate compound produced by gut bacteria for aromatic amino acid synthesis. 4EPS: a gut bacteria derived compound that regulates brain function and cause mental disorders.

Based on the two examples just discussed, we think that more riboswitch-like protein-dependent or even small compound-dependent RNA switches may exist in mammals. Specific structures of potential mammalian RNA switches will “switch” upon binding to a regulatory molecule, such as an RNA-binding protein/complex, a natural metabolite, or a medicinal chemical compound. The structural remodeling in human RNA switches may alter noncoding RNA functions (e.g., mRNA UTR, lncRNA), mRNA stability, or translatability, leading to gene regulation and pathophysiological consequences. We speculate that there will be four types of RNA switches in humans (Fig. 3). We will discuss in detail the rationale for each and possible directions for future research.

### 3.1 Potential RNA switches in mammals mediated by RNA-binding, metabolic enzymes

PDRSs in bacteria, such as the PyrR regulator RNA/protein complex, are well-characterized. PyrR protein is a transcriptional repressor of the pyr operon with uracil phosphoribosyltransferase enzymatic activity. The interaction of PyrR with nucleotide metabolites UMP/UTP determines whether it interacts with a structured RNA regulator in the *pyr* 5′-leader region and stabilizes the conformation of an antiantiterminator stem-loop [29]. For mammalian cells, Dr. Hentz’s laboratory has identified a large number of metabolic enzymes that serve as mRNA-binding proteins via mRNA interactome capture [82], [83], [84]. EPRS is a well-established mRNA-binding metabolic enzyme, as confirmed by these high throughput proteomic screens. Proof-of-principle supporting evidence for this type of RNA-binding, metabolic enzyme-mediated, RNA switch comes from the GAIT and HILDA complex-mediated VEGFA RNA switch, described in the first section (Fig. 1) [21], [23]. The moonlighting mRNA-binding function of EPRS in the GAIT complex maintains the off switch of *VEGFA* mRNA translation, while hnRNP L in the HILDA complex activates the *VEGFA* mRNA translation through the RNA structural switch. Many genes encoding RNA-binding metabolic enzymes have been identified as genes containing genetic mutations that contribute to the pathogenesis in specific human diseases [85]. However, the relation between the RNA-binding ability of these mutated enzymes and pathogenic mechanisms is not fully understood.

In all eukaryotic organisms mitochondrial RNA metabolism is a critical process for synthesizing the component proteins of the respiratory electron transport chain (ETC) complex. mRNA interactome capture, followed by mass spectrometry analysis, has identified a group of noncanonical RBPs in the FAS-activated serine/threonine kinase (FASTK) family of proteins [86]. Among those RBPs, FASTKD2 interacts with select mitochondrial transcripts such as 16S rRNA (RNR2) and NADH dehydrogenase subunit 6 (ND6) mRNA. Genetic deletion of FASTKD2 using CRISPR-Cas9 technology causes abnormal maturation and expression of *RNR2* rRNA and *ND6* mRNA, and consequently, a dysfunctional ETC complex that compromises mitochondrial activity. However, the molecular mechanism is still unclear. We expect that more metabolic enzymes from different cellular compartments will be uncovered to function as new RBPs that contain noncanonical RNA-binding domains. These enzymes may bind to specific mRNAs and regulate their translation, such as adenosine kinase (ADK, localized in nucleus or cytoplasm) and aldolase A (ALDOA, localized in the cytoplasm) (Fig. 3) [87]. It is possible that these enzymes may bind to mRNAs that encode proteins in related metabolic pathways, thereby regulating their translation and cell metabolism. Enzyme binding to their target mRNA(s) could depend on the binding of their substrates (e.g., adenosine or ATP, and fructose 1,6-bisphosphate, for ADK and ALDOA, respectively) or product metabolites (e.g., AMP, and glyceraldehyde 3-phosphate, respectively), as suggested previously [87].

This possible new type of metabolic enzyme-mediated RNA aptamer may be a part of a PDRS if a structural rearrangement or a conformational switch occurs. As another example, isocitrate dehydrogenases (including IDH1, IDH2, and IDH3A) have been reported to be RNA-binding proteins in mouse cardiomyocyte cells [83], but their specific RNA targets in cells and regulatory function in RNA metabolism have not been identified (Fig. 3). CLIP-Seq (crosslinking and ribonucleoprotein immunoprecipitation coupled with RNA-Seq) or RIP (ribonucleoprotein IP)-Seq can be performed to determine the mRNA-binding sites of these enzymes and develop an enzyme–RNA interaction atlas. Based on this atlas, one can examine whether the enzyme interacts with specific transcripts via structural binding motifs and whether this interaction will be modulated by other RBPs or miRNAs as a potential RNA switch mechanism. We anticipate that manipulating the expression level of either enzyme, its substrate(s), or product metabolites will regulate metabolism-related gene expression. These studies may provide new insights into a preventive and therapeutic intervention for the treatment of metabolic syndromes.

### 3.2 Potential ribosome-dependent RNA switch in mitochondria of mammalian cells

Dr. Ronald Breaker suggested that potential physiological human riboswitch-like RNA switches, if they exist, may be found for second messengers and other cellular signaling compounds, rather than for common metabolites, such as ATP [15]. In bacteria, the virulence mRNA in *Salmonella* is an ATP-sensing riboswitch under acidic pH conditions [88]. Another example is the orphan *ydaO* RNA motif, which is a well-established cyclic di-AMP-sensing riboswitch that also weakly binds ATP [89]. Numerous RNA regulators of ribosomal proteins are also known in the bacterial genome [28]. Presumably, mitochondria have a highly reduced DNA genome that is likely to lose riboswitch elements compared to their ancestor alpha-proteobacteria. However, purine riboswitches and several ribosomal protein regulators are preserved in the reduced genomes of some symbiotic bacteria [90]. Therefore, we cannot rule out the possibility that mitochondrial mRNAs may still contain potential remnants of *cis*-acting RNA elements to regulate translation efficiency, dependent on either specific RNA-binding proteins or ribosomes. Mitochondria produce the majority of ATP for conducting cellular functions, and the ATP level needs to be fine-controlled. In yeast, mRNAs of mitochondrial-encoded genes (MEGs) contain 5′UTRs, and many regulatory proteins bind to the 5′UTR to modulate MEG translation and ATP production [91], [92]. In contrast, the mammalian mitochondrial genome is a short and compact circular DNA molecule, with either extremely small or no untranslated regions, that encodes for only 13 coding genes and 24 noncoding genes. The 13 coding genes include components of the ETC, such as subunits of NADH dehydrogenase (Complex I), CoQH_2_-cytochrome *c* reductase (Complex III), cytochrome *c* oxidase (Complex IV), and ATP synthase (Complex V). Mammalian mitochondria also lack the homologs of translational regulators that bind to 5′UTR of MEG mRNAs in yeast. So, what is the mechanism for translational control of MEGs in mammalian mitochondria?

**Fig. 4:**
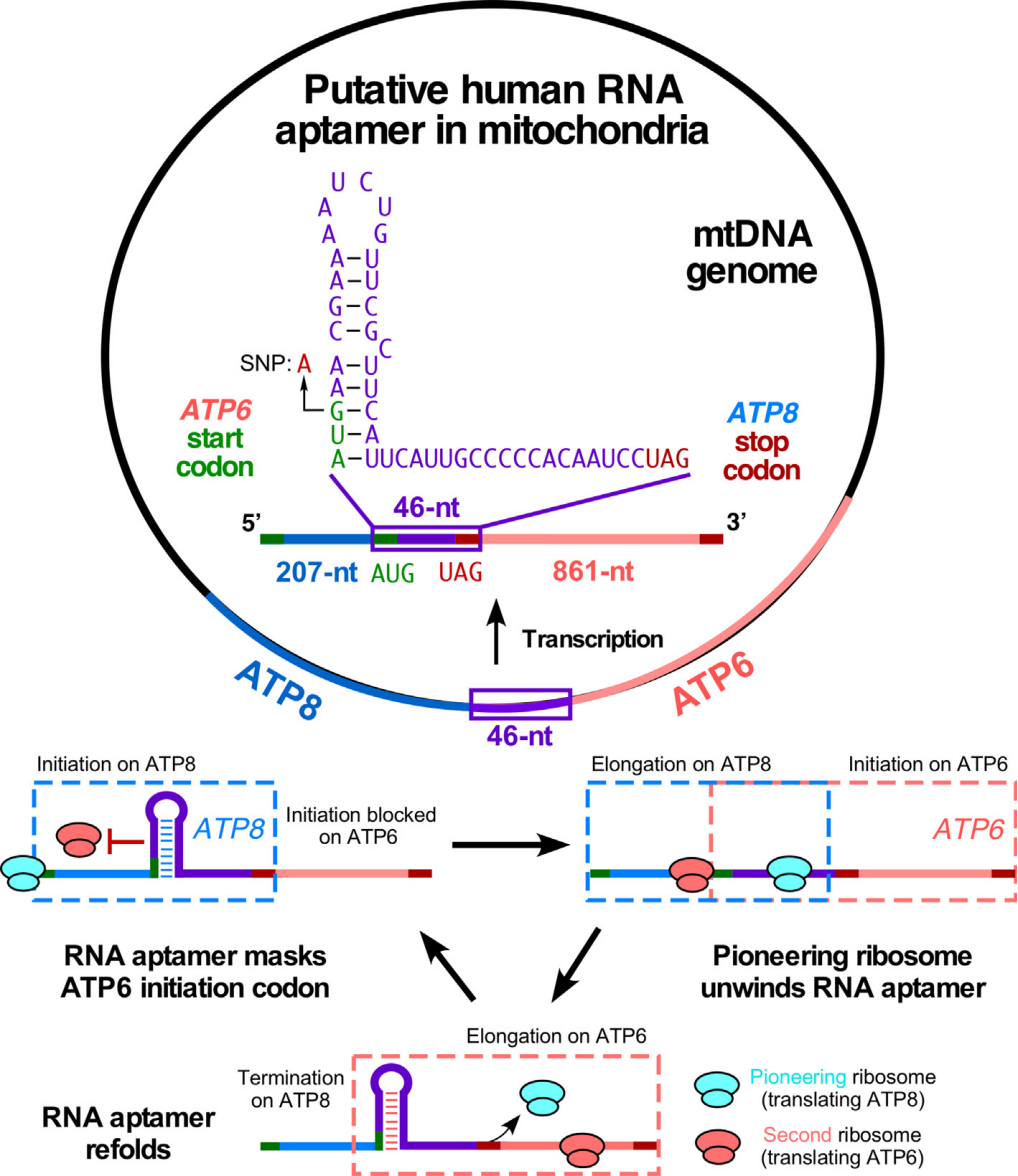
A putative RNA switch element located in mammalian mitochondrial *ATP8/ATP6* mRNA. A 46-nt overlapping region was uncovered in both ATP8 and ATP6 genes. This RNA fragment may form a putative stem-loop structure, which inhibits ribosome loading and translation initiation for ATP6 until the pioneering ATP8-translating ribosome unwinds it to mediate the coordinated translation of ATP8/ATP6 mRNA and maintain 1:1 stoichiometric ratio of both proteins.

A peculiar feature for two gene pairs, ATP8/ATP6 (two MEGs encoding Complex V constituents of the ETC complex) and ND4L/ND4 (two MEGs encoding Complex I constituents of the ETC complex), is that their genes overlap, resulting in a bicistronic transcript with no UTRs. For the former, transcription starts from the upstream gene, ATP8, covering the overlapping region between the two genes, and stopping at the end of a downstream gene, ATP6. This process yields one long mRNA molecule that is eventually translated into two different proteins [78] (Fig. 3, Fig. 4). The underlying reason for this phenomenon is not apparent. The stoichiometric ratio between ATP8 and ATP6 needs to be tightly controlled to enable assembly of F_0_ complex and functional Complex V without producing idle component proteins that could cause mitochondrial unfolded protein stress [93]. The exact mechanism for coupling of the translation of ATP8 and ATP6 is still unknown. One possible clue lies in the overlapping region between the ATP8 and ATP6 genes.

The TurboFold algorithm integrates multiple sequence alignment results from a group of homologous sequences with nearest neighbor base pairing thermodynamics, to predict most probable RNA secondary structures [94]. Running this algorithm on human, chimpanzee, mouse, and sheep sequences of ATP8 shows a putative hairpin loop that starts almost around the ATP6 start codon AUG (Fig. S1). This same predicted hairpin appears in many of the sequences applied from other mammalian species (not shown). For instance, an overlapped 46-nt RNA region is present in the coding sequence of the human MT-ATP8/ATP6 bicistronic mRNA. This region is located in *ATP6*, beginning with AUG start codon, and *ATP8* ending with UAG stop codon with +1 nt frameshift. The 46-nt RNA region may form a stem-loop structure (mfold web server) [95] containing a 6-nt loop, a 9-bp stem, and a single C bulge. The structure is consistent with the prediction from the TurboFold algorithm (Fig. 4). The mouse RNA aptamer contains two wobble base pairs, while the human RNA aptamer contains all nine Watson-Crick base pairs.

We hypothesize that this 46-nt RNA element may contribute to translational regulation of the stoichiometry of ATP8 and ATP6 proteins in a ribosome-dependent manner (Fig. 3). Since mitoribosome and Complex V are close in inner membranes, the 46-nt RNA secondary structure could inhibit ribosome loading and translation initiation for *ATP6* by sequestering the AUG start codon. The pioneering ribosome translating ATP8 may unwind the stem-loop structure and allow a second ribosome to translate ATP6 as a coupling mechanism, to ensure the 1:1 stoichiometric ratio of ATP8 and ATP6 (Fig. 4). Therefore, the assembly of F_0_ complex into Complex V is coordinated to maintain homeostatic ATP production. The stem-loop may re-form after the first ribosome reads through the region and prevents another ribosome from translating ATP6 by masking its initiation codon AUG. It is likely that the mitoribosome acts as an RNA helicase involved in the regulation of translation efficiency of its bound mRNA, in the absence of a counterpart of the cytosolic DDX proteins. We cannot completely rule out the possibility that this ATP8/6 RNA aptamer is bound by a mitochondria-localized RNA-binding protein or a specific mitochondrial metabolite (e.g., ATP) for regulating mRNA translation [96], [97], although the likelihood is low. Intriguingly, multiple SNPs were found located in the stem region within or adjacent to the AUG start codon at the 5′-end of the putative 46-nt RNA aptamer (dbSNP). One synonymous SNP changes AUG to AUA (both are codons for Met in ATP6) and may impair the stability of the stem region by disrupting a G-C Watson-Crick base pair (Fig. 4). This specific SNP could potentially influence the structure and function of the putative human mitochondrial ATP8/6 regulatory RNA switch element. In genome-wide association studies, this type of SNP-mediated RNA-based regulatory mechanism has been found in 5′UTR and 3′UTR of mRNAs and lncRNAs as riboSNitch [98], [99].

### 3.3 Potential mammalian RNA switches that depend on pathogenic metabolites

Prokaryotic and eukaryotic cells have well-established metabolic sensing systems that are important for cell survival and environmental adaptations [6], [100]. As a complicated integration of trillions of cells, the human body has the multiplex biological buildup of cellular metabolisms across a variety of organs and within multiple cell types in each organ. The metabolic pathways are highly interconnected by refined metabolite-sensing mechanisms to a biological network that comprises a broad spectrum of metabolites, metabolic enzymes, and membrane-bound receptors [100], [101]. It has been documented that among these many macromolecules, RNA plays a critical regulatory role in cell metabolism and environment sensing in response to exogenous metabolites [102], [103], [104]. As one example, aminoglycoside antibiotics isolated from bacteria, such as streptomycin and neomycin, have been proven to specifically target the rRNA aminoacyl A-site of the pathogenic microorganisms in infected human patients [105]. However, it is still unclear whether small chemical compounds in the environment influence the structure and activity of RNA from human cells. The gut microbiota contains more microbial cells than all the human cells in the body and functions as a symbiotic endocrine organ [106], [107], [108]. It produces unique physiologic and pathogenic metabolites that affect host health and may provide the environment to evolve host cell riboswitch-like RNA aptamers activated by a paracrine ligand produced in bacteria. We hypothesize that pathogenic RNA switches could be mediated by metabolites generated from gut microbiota (e.g., trimethylamine N-oxide, chorismic acid, 4EPS) or environmental factors (e.g., nicotine) (Fig. 3). These potential RNA switches may be involved in human disease etiology and pathogenesis.

Dr. Hazen’s laboratory has reported that the metabolite, trimethylamine N-oxide (TMAO), but not its precursor trimethylamine (TMA), triggers cardiovascular disease (CVD) in both mice and humans [106], [109], [110]. Dietary choline (or l-carnitine) from red meat is first converted into TMA by gut microbiota and then oxidized to TMAO by the hepatic enzyme, flavin-containing monooxygenase 3 (FMO3), and excreted into the circulation. Based on several clinical reports, the TMAO level in the circulatory system could be an important clinical biomarker for evaluating a patient’s risk for major adverse cardiovascular events [111], [112]. One recent study reported that plasma TMAO levels could be considered a novel biomarker for plaque rupture in myocardial infarction patients with ST-segment elevation [113]. CD36 and SR-A, two scavenger receptors on the surface of macrophages, bind oxidized low-density lipoprotein (oxLDL) to promote foam-cell formation and atherogenesis. The levels of these receptors are significantly upregulated in macrophages of TMAO-treated mice [106], suggesting that TMAO could serve as a clinical biomarker and therapeutic target for the prevention and treatment of CVD [106], [109], [110]. However, the molecular mechanisms underlying the pathogenic role of TMAO and TMAO-triggered upregulation of CD36 and SR-A remain unresolved.

A high concentration of TMAO is known to stabilize the tertiary structure of tRNA^fMet^, but does not affect its secondary structure [114]. TMAO also facilitates the formation of the native structure of multiple RNAs, as does Mg^2+^ [115]. We hypothesize that a pathological level of TMAO (∼100 μM) may induce transcript-specific mRNA structural remodeling in host cells in a paracrine manner, altering the accessibility of miRNAs or RBPs to mRNA 3′UTR (e.g., CD36, SR-A), and thereby modifying the cellular transcriptome or translatome*.* As a consequence, changes in the translatome landscape may contribute to the progression of multiple CVDs promoted by TMAO (e.g., atherosclerosis, heart failure). It has recently been reported that the levels of miR-146-5p and its targets NUMB and DLST are strongly linked to TMAO levels and may be involved in the progression of atherosclerosis [116]. The potential interaction of TMAO with either the miR-146-5p precursor or downstream target mRNAs NUMB and DLST could be further examined to test the hypothesis of TMAO–RNA interaction.

### 3.4 Potential mammalian RNA switches driven by medicinal compounds

Metabolite analogs and small chemical compounds have been screened as possible inhibitors of bacterial riboswitches, to assess their potential as candidates for the development of antibiotics [117], [118]. In addition, it has recently been reported that a growing list of medicinal chemical compounds can bind to specific human cellular or viral RNAs and influence their structures or functions in human cells [119], [120], [121], [122], [123]. Such therapeutic compounds are promising to consider as next-generation RNA-targeted therapeutics for treating human diseases, ranging from myotonic dystrophy to viral infection to cancer [119], [120], [121], [122], [123].

For example, a benzimidazole (1) compound selectively binds to the precursor stem-loop structure of miR-96 and inhibits its processing and maturation, thereby inducing cancer cell apoptosis [122]. These findings indicate that noncoding RNA structural elements in mammals may be targeted by small synthetic molecules. A large literature addresses the transcriptome-wide expression profile of cell lines under treatment by a specific chemical compound, such as ibuprofen, atorvastatin, and aspirin [124], [125], [126]. However, less attention has been paid to posttranscriptional regulation at the level of protein expression. Interactions between chemically synthesized drugs and cellular RNAs have been sparsely studied and reported. However, in one study, FDA approved anti-cancer drugs (e.g., kinase and topoisomerase inhibitors) were found to bind the oncogenic precursor of miR-21 and inhibit its maturation and expression [127]. This drug–RNA interaction may change the stability and translation efficiency of downstream target mRNAs of miR-21, which might partly account for a therapeutic consequence, drug resistance, or even side effects in multiple human diseases including cancer and CVD [128], [129], [130].

Drug–RNA interactions need to be investigated for popular medications, such as ibuprofen, aspirin, statin, metformin, and many other frequently prescribed medicines. We propose that potential pharmacological RNA switching may be mediated by some of these human-manufactured chemical compounds (Fig. 3). For instance, the Precision (Prospective Evaluation of Celecoxib Integrated Safety versus Ibuprofen or Naproxen) Trial reported the comparative cardiovascular safety of three non-steroidal anti-inflammatory drugs [131]. Serious concerns have been raised about this 10-year clinic trial involving over 24,000 human subjects [132]. Considering the undesirable side-effects of the three drugs in triggering major adverse cardiovascular events, the molecular mechanisms of which remain unknown, the possibility of inappropriate dosages and potentially dangerous interactions with aspirin have been voiced. Pharmaceutical companies have rigorously examined drug–drug and drug–protein interactions, but it is not certain whether FDA-approved drugs may interact with specific cellular RNAs and influence the transcriptome-wide translational profile. We surmise that medicinal chemical compounds may bind to specific cellular mRNAs or noncoding RNAs, and these potential drug–RNA interactions could modulate RNA expression, processing, and function, or alter protein synthesis by changing the RNA structure*.* These putative effects could explain the hormetic (i.e., biphasic) activity of most therapeutic compounds, partly accounting for drug side-effects. These potential drug–RNA interactions might also underlie the therapeutic benefits of some drugs (e.g., riboswitch-targeted antibiotics and cellular RNA-targeted compounds), or the development of drug-resistance (e.g., anti-cancer medicines that may inactivate maturation of a tumor suppressor miRNA).

## 4 Concluding remarks

A riboswitch is a critical component of the RNA world. A spectrum of roles of mammalian RNA switches, which resemble riboswitches and riboswitch-like mechanisms in prokaryotes and simpler eukaryotes, have been uncovered over the last two decades in microbiology and cellular biology studies. Cellular stresses activate or inhibit protein-directed RNA switches under pathogenic conditions. In mammals, the principal executors of RNA switching are RNA-binding proteins and miRNAs. The mammalian PDRSs and potential riboswitch-like, metabolite-dependent RNA switches may have four unique properties: (a) They are not necessarily conserved in evolution from bacteria to eukaryotes and could be present in genes from particular species under specific conditions, such as the VEGFA RNA switch. (b) Unlike most bacterial riboswitches, which are unique to a single transcript, human RNA switches can respond to a protein ligand or a metabolite and regulate a cohort of transcripts that provide cells with the ability to adapt to changing environments. (c) Previous findings indicate that human PDRSs confer a 2–5-fold change in gene expression, which is less than the 10-fold change found in bacterial riboswitches, but on par with the action of other human regulatory mechanisms, such as those mediated by miRNAs, that likely function in parallel with other regulatory factors to fine-tune gene expression. (d) We anticipate that human RNA switches are not restricted to mRNA 5′UTR but are also present in mRNA coding regions (e.g., ATP8/ATP6) and other untranslated RNAs (e.g., *CD36* 3′UTR, lncRNAs).

Several questions need elucidation to clarify the molecular mechanism of PDRSs in general. These include: (a) How widely is PDRS distributed in different animal species and across distinct cell types in each species? (b) What are the global targets of PDRSs, including mRNAs and noncoding RNAs? (c) What is the relevance of PDRSs in human disease and medicine? Although a variety of PDRSs has been convincingly shown, it is unclear whether mammalian riboswitch-like RNA switches that depend on metabolites are present or not. To resolve these questions, we need a hypothesis-driven investigation (Fig. 3, Fig. 4) or unbiased broad-scale and high-throughput screening studies [119], [120] that include two-dimensional combinatorial screening (2DCS) and chemical crosslinking and isolation by pull-down (Chem-CLIP). These investigations should be combined with experimental, observation-driven, target-specific validation studies.

We propose that defects in putative human RNA switches contribute to both normal and disease-associated changes of gene expression and cellular metabolism. A better understanding of the molecular mechanisms of human RNA switches, which could be physiologic, pathogenic or pharmacological, will improve and promote the rational design of drugs that target RNA switches for treatment, and may help to reduce the side-effects of medication. We predict that new research fields will be created to elucidate the interplay between small molecules and RNAs in human health and disease (Fig. 3). Understanding the molecular mechanisms that underlie human RNA switches will clarify their roles in normal and pathological cellular processes and contribute to the development of new diagnostic and treatment strategies for human disease.

## Declaration of Competing Interest

The authors declare that they have no known competing financial interests or personal relationships that could have appeared to influence the work reported in this paper.
