# Supplementary material for: Mammalian RNA switches: Molecular rheostats in gene regulation, disease, and medicine
Source: Comput Struct Biotechnol J. 2019 Oct 24;17:1326–38. doi: 10.1016/j.csbj.2019.10.001 (PMC6849081; doi:10.1016/j.csbj.2019.10.001)
Supplement: Supplementary data 1 [file mmc1.docx]

**Fig. S1**. cDNA sequences and TurboFold algorithm-predicted structures of potential ATP8/ATP6 RNA aptamers from human, chimpanzee, mouse, and sheep. The putative RNA element in ATP8 is highlighted by a yellow box in the predicted structure. The sequence of the RNA element is marked in yellow to align with structural annotation.

**
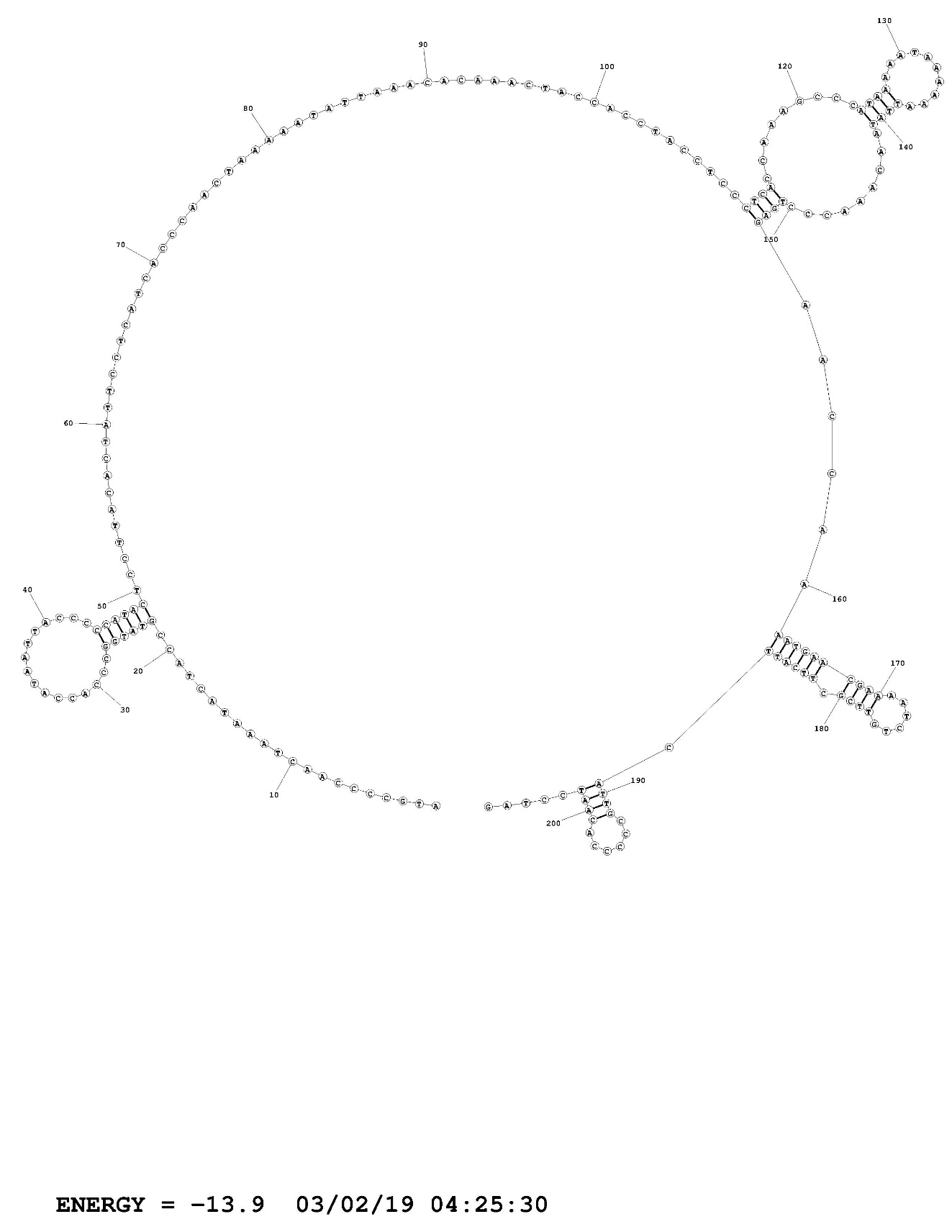
**

46-nt RNA element

Predicted structure of putative human ATP8/ATP6 RNA aptamer (nt 162-207)

Human ATP8 cDNA sequence:

**>NC_012920.1:8366-8572 Homo sapiens mitochondrion, complete genome**

ATGCCCCAACTAAATACTACCGTATGGCCCACCATAATTACCCCCATACTCCTTACACTATTCCTCATCA

CCCAACTAAAAATATTAAACACAAACTACCACCTACCTCCCTCACCAAAGCCCATAAAAATAAAAAATTA

TAACAAACCCTGAGAACCAAAATGAACGAAAATCTGTTCGCTTCATTCATTGCCCCCACAATCCTAG

**
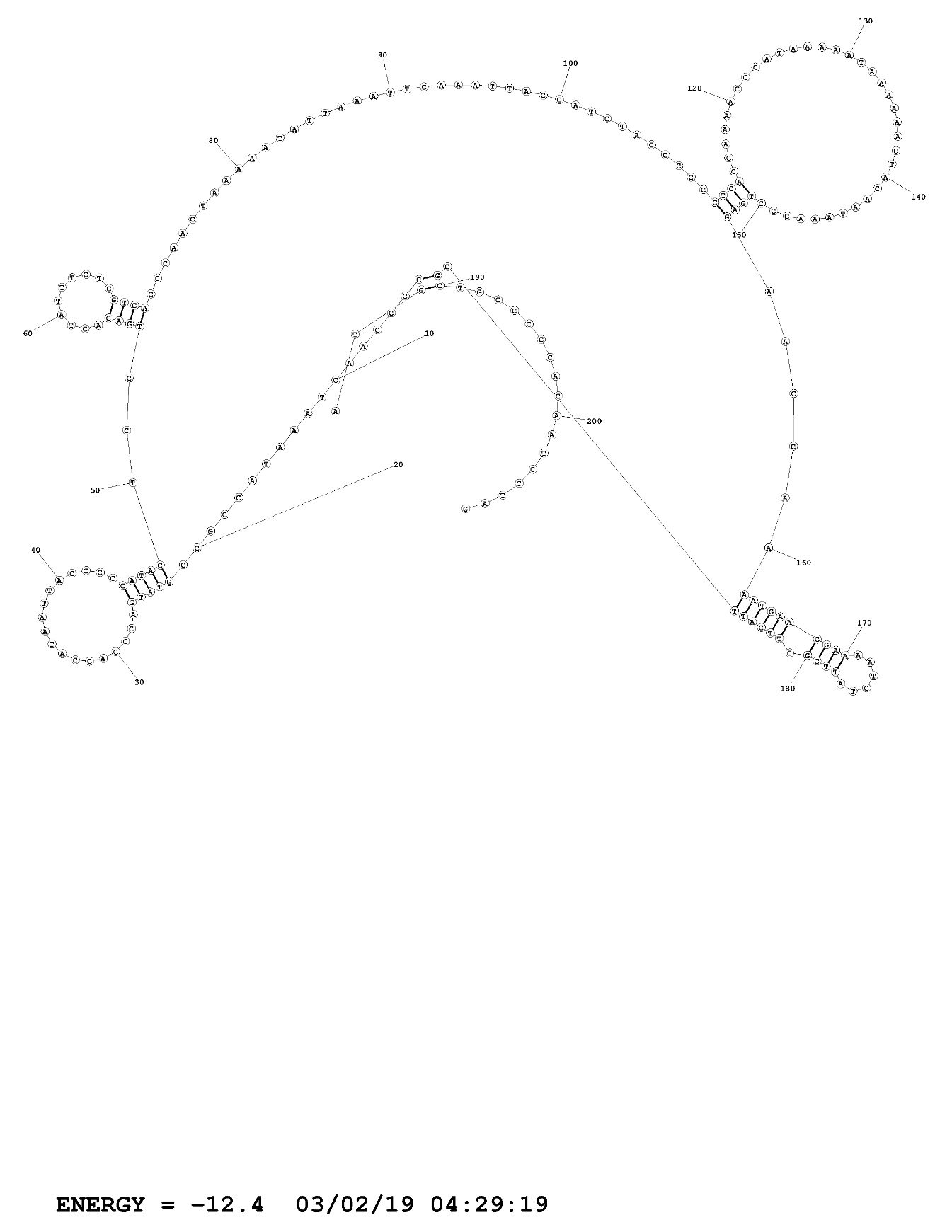
**

46-nt RNA element

Predicted structure of putative chimpanzee ATP8/ATP6 RNA aptamer (nt 162-207)

Chimpanzee ATP8 cDNA sequence:

**>NC_001643.1:7784-7990 Pan troglodytes mitochondrion, complete genome**

ATGCCCCAACTAAATACCGCCGTATGACCCACCATAATTACCCCCATACTCCTGACACTATTTCTCGTCA

CCCAACTAAAAATATTAAATTCAAATTACCATCTACCCCCCTCACCAAAACCCATAAAAATAAAAAACTA

CAATAAACCCTGAGAACCAAAATGAACGAAAATCTATTCGCTTCATTCGCTGCCCCCACAATCCTAG

**
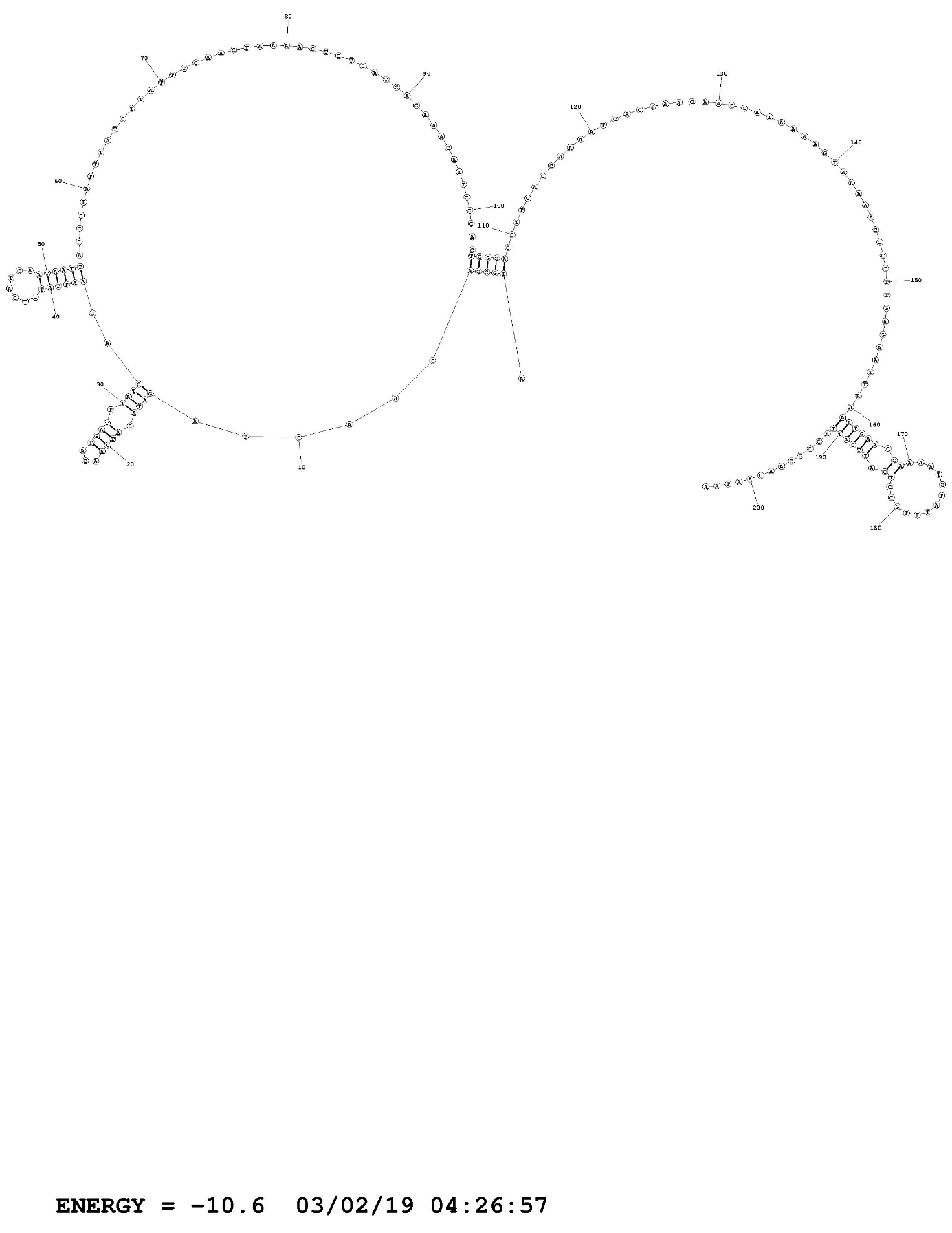
**

43-nt RNA element

Predicted structure of putative mouse ATP8/ATP6 RNA aptamer (nt 162-204)

Mouse ATP8 cDNA sequence:

**>NC_005089.1:7766-7969 Mus musculus mitochondrion, complete genome**

ATGCCACAACTAGATACATCAACATGATTTATCACAATTATCTCATCAATAATTACCCTATTTATCTTAT

TTCAACTAAAAGTCTCATCACAAACATTCCCACTGGCACCTTCACCAAAATCACTAACAACCATAAAAGT

AAAAACCCCTTGAGAATTAAAATGAACGAAAATCTATTTGCCTCATTCATTACCCCAACAATAA

**
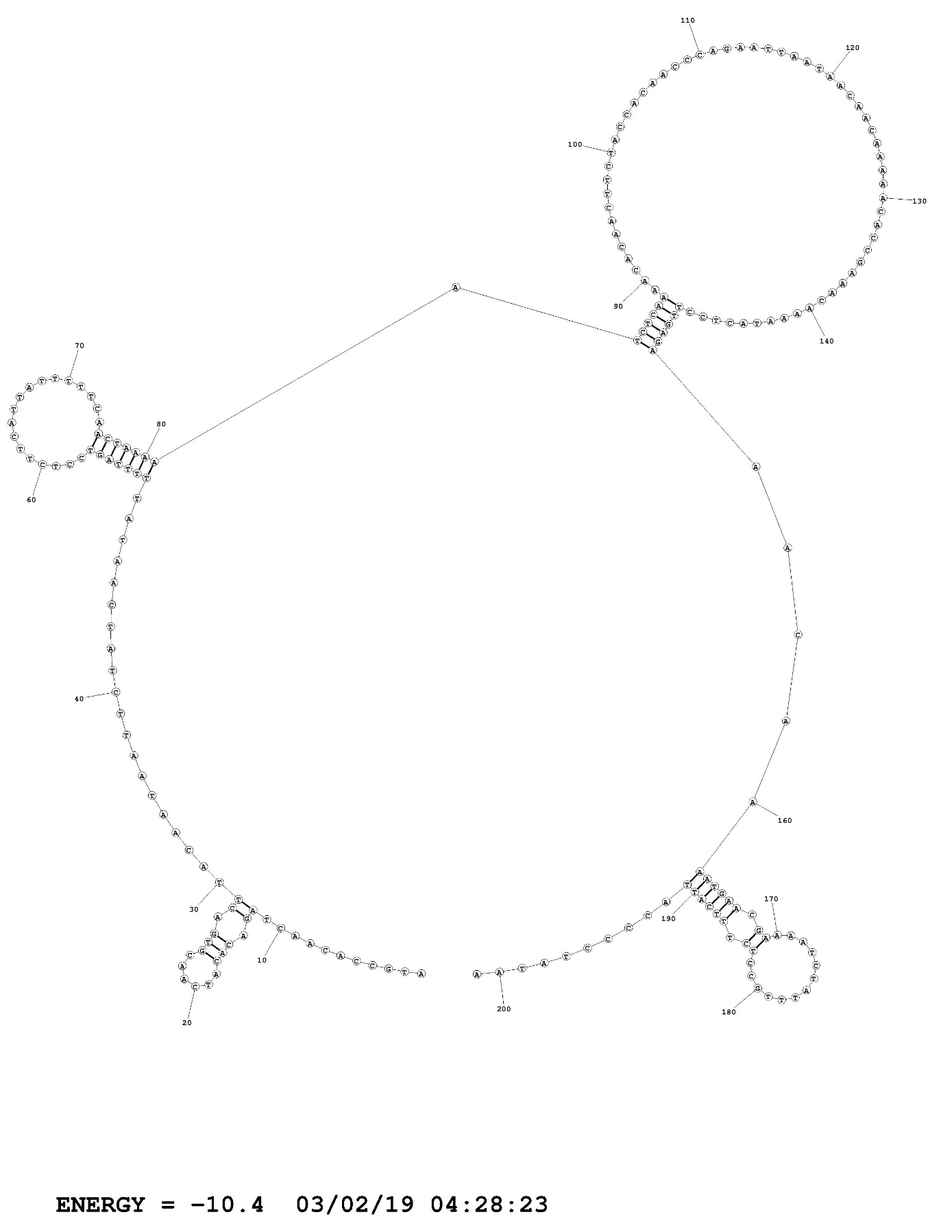
**

40-nt RNA element

Predicted structure of putative sheep ATP8/ATP6 RNA aptamer (nt 162-201)

Sheep ATP8 cDNA sequence:

**>NC_001941.1:7775-7975 Ovis aries mitochondrion, complete genome**

ATGCCACAACTAGACACATCAACGTGACTTACAATAATTCTATCAATATTTTTAGTCCTCTTCATTATTT

TTCAACTAAAAATCTCAAAACACAACTTCTACCACAACCCAGAATTAATAACAACAAAAACACCGAAACA

AAATACTCCTTGAGAAACAAAATGAACGAAAATCTATTTGCCTCTTTCATTACCCCTATAA
